# Emerging Processes for Sustainable Processing of Food Ingredients and Products

**DOI:** 10.3390/foods12193633

**Published:** 2023-09-30

**Authors:** Roberto Castro-Muñoz

**Affiliations:** 1 Tecnologico de Monterrey, Campus Toluca, Avenida Eduardo Monroy Cárdenas 2000 San Antonio Buenavista, Toluca de Lerdo 50110, Mexico; food.biotechnology88@gmail.com; 2 Department of Sanitary Engineering, Faculty of Civil and Environmental Engineering, Gdansk University of Technology, 11/12 Narutowicza St., 80-233 Gdansk, Poland

## 1. Introduction 

In recent decades, traditional food processing processes, such as homogenization, pasteurization, canning, drying, and smoking, among others, have been successfully applied to obtain, to some extent, acceptable food items. However, with the increasing food demand, as a consequence of the growing population worldwide, new, tunable, and enriched food products are demanded, requiring the implementation of emerging technologies in different areas of food processing. Such technologies offer the possibility of tuning the properties of food ingredients and several products and byproducts from traditional transformation processes [1,2]. Additionally, innovative technologies are providing relevant insights regarding reducing their waste, representing a promising alternative to environmental issues as well [3]. Therefore, we point out the importance of innovative and emerging techniques for processing food ingredients, products, and new food formulations.

## 2. Biorefining or Upgrading of Food Ingredients

As part of the increasing demand of food ingredients aimed at food fabrication, new sources of bioactive compounds and nutraceuticals are pursued. At this point, not only natural sources but also the main agrifood by-products and wastes (waste waters, pomace, seeds, skin, leaves, liquors, roots, fibers, etc.) become popular as potential sources of food ingredients [4,5]. However, the complete characterization of these sources is challenging since plenty of other compounds are found in natural sources. Therefore, the implementation of adequate extraction, separation, fractionation, and polishing of the target metabolites is challenging; however, emerging techniques, such as membrane processes, ultrasound-assisted extraction (UAE), microwave-assisted extraction (MAE), pressurized liquid extraction (PLE), supercritical fluid extraction (SFE), pulsed electric field (PEF), moderate electric field (MEF), and high-voltage electrical discharge (HVED), have been implemented successfully towards the recovery task [6,7]. After successful extraction and separation, adsorption and chromatographic techniques, as high-performance purification techniques, are the most suitable for obtaining high-purity compounds. Therefore, this is current research topic undergoing extensive exploration by the research community. 

## 3. Tuning Physiochemical Properties of Food Products and Bio-Products

Today, according to the necessity of consumers, food manufacturers are in need of tunning the specific physicochemical properties of food products, such as hydration properties (water activity, water absorption capacity, water retention capacity, hygroscopicity, dispersibility, solubility, etc.), rheological fluid behavior, mechanical properties, optical properties (color, translucence, etc.), and thermal and organoleptic (taste, texture, mouthfeel, aroma) properties [8]. Of course, the tunning of the physicochemical properties will dictate the final properties of the elaborated product. Therefore, the current scope of research deals with the application of traditional and innovative technologies for tunning the physicochemical properties of food items. For instance, in the latter field, hydrodynamic cavitation, ultrasound, microwave-assisted processes [9] are some examples of current applied techniques for the modification of proteins, carbohydrates, nano-emulsions, fats, and bioactive compounds (carotenes), among many others.

## 4. Process Intensification in Food Processing

As in several research and industrial fields, process intensification relies on the design of novel food processing equipment and their coupling to other unit operations in order to improve food production capacity, selectivity, efficiency, cost, and reduced energy consumption and wastes. Usually, the exploration of integrated processing technologies is carried out to more efficiently obtain a food production process and products with enhanced quality. For instance, acoustic cavitation, hydrodynamic cavitation, membrane processes (ultrafiltration, pervaporation, membrane distillation), and enzymatic treatment are some of the processes and technologies introduced during the process intensification of oils, juices, beverages, and dairy products. 

## 5. Reduction in Undesired Compounds from Food Systems into the Environment

Today, as part of the environmental regulation and green chemistry principles, there is a current need to reduce the production of by-products and wastes derived from food processing processes. In recent decades, such wastes with high organic composition were directly verted into the environment, causing sever issues in water bodies and ecosystems. In some countries, the reduction in wastes (olive mill wastewater, grape pomace, artichoke wastewater, among others) has been substantially reduced through the valorization of such residues, proposing them as a new feedstock for obtaining food ingredients, including hydroxycinnamic acids, carotenoids, flavonoids, capsaicin, and gingerols, among many other bioactive molecules. Herein, the current scope of research deals with the investigation of agro-food wastes as potential sources of bioactive compounds.

Therefore, this Special Issue points out the importance of innovative and emerging techniques for processing food ingredients, products, new food formulations, and their resulting byproducts and wastes. It welcomes both original and compelling review contributions related to such applications and cases of studies using emerging processes of high-added-value and non-desired compounds for possible interest in food industries. These topics are the main interests of this Special Issue, but we are not limited to them.
